# Exosome-Induced Regulation in Inflammatory Bowel Disease

**DOI:** 10.3389/fimmu.2019.01464

**Published:** 2019-06-28

**Authors:** Huiting Zhang, Liang Wang, Changyi Li, Yue Yu, Yanlin Yi, Jingyu Wang, Dapeng Chen

**Affiliations:** ^1^Comparative Medicine Department, Dalian Medical University, Dalian, China; ^2^Laboratory Animal Center, Dalian Medical University, Dalian, China

**Keywords:** exosome, inflammatory bowel disease, inflammation, immunology, intestine

## Abstract

An exosome (30–150 nm size) is a cell-derived vesicle. Exosome-induced regulation in inflammatory bowel disease (IBD) is becoming increasingly popular due to their potential functions of exosomal pathways. Exosomes, which are involved in the regulation of IBD, can be released from various cell types, or found in many physiological fluids, and plants. The specific functions of exosomes in IBD primarily depend on the internal functional components, including RNAs, proteins, and other substances. However, exosome-induced transport mechanisms involving cell-cell communications or cell-environment interactions are also very important. Recent studies have revealed that exosome crosstalk mechanisms may influence major IBD-related pathways, such as immune responses, barrier functions, and intestinal flora. This review highlights the advancements in the biology of exosome secretions and their regulation in IBD. The functional roles of exosomal components, including nucleic acids, proteins, and some other components, are the main focus of this review. More animal and clinical research is needed to study the functions of exosomes on IBD. Designing new drug dosage form using exosome-like-structure may provide new insights into IBD treatment. This review suggests a potential significance for exosomes in IBD diagnosis and treatment.

## Introduction

Inflammatory bowel diseases (IBD) are a family of chronic autoimmune diseases of the gastrointestinal tract, including Crohn's disease (CD) and ulcerative colitis (UC) (1). Symptoms can be characterized as intermittent recurrence and quiescent inflammatory remission (2). Incidence rates of CD and UC vary between 0.1–11 and 0.5–24.5 per 100,000 people, respectively, in various geographic areas around the world (3). The annual recurrence rate of IBD in China is between 0.0145 and 0.0196%. Young and middle-aged patients may suffer from severe complications, and thus, impose heavy economic burdens both on their families and on society (4).

The pathogenesis of IBD remains under studied. Likely, pathogenic mechanisms of IBD comprise of environmental or genetic factors, gut microbiota and immune responses (5, 6). The intestinal microbiota is associated with the development and maintenance of IBD, and fecal microbiota transplantation from healthy controls to IBD patients has been used clinically as an emerging treatment for IBD (7). Gut microbiota-derived extracellular vesicles (EVs) are worthy of further research as they have the potential to uncover mechanisms underlying fecal microbiota transplantation-induced treatment effects and limitations of this treatment (7, 8). Many systematic reviews of EVs have been completed in recent years and have predominantly found EVs as modulators in many diseases, including diabetes, cardiovascular disease, coagulopathies, polycystic ovary syndrome, and autoimmune diseases (9–11).

**Figure 1:**
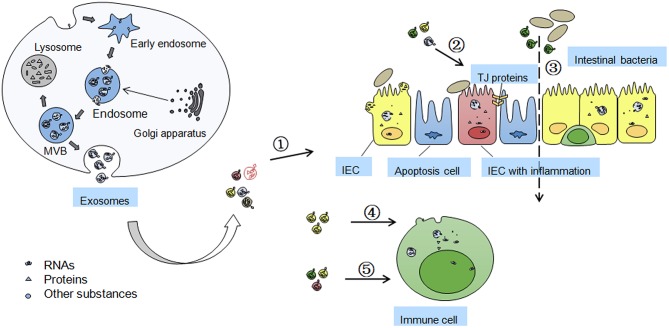
Formation of exosomes and functions/mechanisms of exosomes in IBD. Exosomes are formed by inward budding of cell membrane and are released through fusion of multi-vesicular body (MVB) with the membrane. MVB can be degraded by fusion with lysosome and losing the activity of components inside. Golgi apparatus in origin cells can accumulate some exosomal proteins before their secreting into the exosomes. The effects of secreted exosomes on IBD includes ➀ the exosomes isolated from serum and saliva may be novel biomarkers of IBD; ➁ Exosomes from colon cancer cells, IECs, and platelets, can maintain TJ barrier function; ➂ Exosomes from DCs may influence intestinal microbiota profile in heat shock proteins dependent manner; ➃ Intestinal epithelial cell (IEC)-derived exosomes can fuse with the membrane of dendritic cells (DCs) to induce immune tolerance; ➄ Exosomes from colitis serum or treated DCs regulates immune cell proliferation through MAPK, NF-κB, and other inflammation related signaling pathways.

As shown in Figure 1, EVs, sometimes distinguished by size into exosomes or microparticles, are membrane enclosed particles released from many cell types (12). Exosomes are small membrane vesicles (30–150 nm size) released from most cells into the extracellular space that contain various biological components, such as nucleic acids (mRNAs, microRNAs (miRNAs) etc.), proteins, and other components (13, 14).

Exosomes can be found in almost all living cells, especially in dendritic cells (DCs), lymphocytes, epithelial cells (ECs), and endothelial cells. Exosomes can also be detected and obtained from various body fluids such as blood, urine, saliva, amniotic fluid, and breast milk (15). The known origins of exosomes are introduced below.

## Serum and Saliva Exosomes in IBD

In the study by Wong et al. the serum exosomes in acute colitis mice are isolated and studied (16). Proteomic analysis identified 56 proteins, most of which are acute-phase proteins and immunoglobulins. It is also found that the serum exosomes can significantly activate macrophages *in vitro*. This study indicates the potential for serum exosomes in the diagnosis of IBD.

In the study by Zheng et al. exosomes were extracted from saliva of IBD patients (17). More than 2,000 proteins were detected in exosomes from IBD patients, far more than that in healthy people. Eight of them were focused on, most of which were related to inflammation, proteasomes activity, and immune response. Gene ontology analysis showed that proteasome subunit alpha type 7 (PSMA7) was expressed at much higher levels in patients with IBD compared with those in healthy controls. This study indicates an ideal biomarker for IBD diagnosis.

## Exosomes From Cells, Physiological Fluids, and Plants

Many exosomes are not detected in IBD patients or animal models, however, the components contained in exosomes have potential regulatory effects on IBD, and the sources of these kinds of exosomes were listed in Supplementary Table 1.

### Exosomes From DCs

DCs, the most effective or “professional” of the antigen presenting cells located at surveillance sites, have the ability to capture, and process antigens to initiate primary immune responses (18). Depending on the type and stage of maturation of the DCs, DC-derived exosomes have been shown to possess immunostimulatory or suppressive effects. Mature DC-derived exosomes carrying tumor antigens could effectively induce anti-tumor immunity *in vitro* trials, whilst immature exosomes demonstrated their involvement in T-cell immunosuppression to induce peripheral tolerance (19). Moreover, *in vitro* studies have indicated that immunosuppressive gene transforming growth factor (TGF)-β1-modified DCs can produce exosomes to attenuate Th17-mediated dextran sulfate sodium (DSS)-induced IBD by inducing regulatory T cells (mainly CD4^+^Foxp3^+^Tregs) from mesenteric lymph nodes of the inflammatory site (20). Exosomes derived from DCs treated with interleukin-10 (IL-10) or transfected with the gene for IL-10 can inhibit trinitrobenzene sulfonic acid (TNBS)-induced rat colitis through stimulating CD4^+^CD25^+^Tregs (19). It is conceivable that exosomes may be used as vehicles for the therapeutic intervention of autoimmune diseases (20).

### Exosomes From Intestinal Epithelial Cells (IECs)

As a crucial role of the intestinal barrier, IECs can protect against dietary antigens or other harmful substances like viruses and bacteria from food. Thus, IECs can separate external and internal compartments, and exosomes isolated from IECs are pivotal for IEC-induced immune tolerance (14, 21). The IEC-derived exosome-induced immune tolerance depends on internal components like proteins and miRNAs. IEC-derived exosomes might contain high levels of major histocompatibility complex (MHC) I and II molecules in inflammation associated with MHC II-dependent antigen-specific tolerance, which may directly affect IBD conditions (22). Besides, potential exosomes from IEC-6 containing miR150 were isolated, and functions of miR150 in IBD inflammation were also studied.

### Exosomes From Other Cells

Exosomes secreted by mesenchymal stem cells are able to self-renew and can repair tissue injury diseases. Furthermore, exosomes released from human umbilical cord mesenchymal stem cells have been shown to exert their impact through the inhibition of higher IL-7 expression in macrophages to alleviate DSS-induced IBD (23). There are also functional exosomes from another type of cell called granulocytic myeloid-derived suppressor cell. Known as CD11b^+^Ly6G^+^Ly6C^low^ cells with granulocyte-like morphology, they act as major populations of myeloid-derived suppressor cells (24). Exosomes from granulocytic myeloid-derived suppressor cell could alleviate DSS-induced colitis through inhibiting Th1 cells proliferation and promoting Tregs expansion (25).

Besides, exosomes from other cells, like krüppel-like factor 5-overexpressing vascular smooth muscle cells (26–28), human leukocyte antigen-G1-transfected cells (29, 30), myeloid-derived suppressor cells (31, 32) or transglutaminase type 2 proficient cells (33, 34), were studied, while the contents in these exosomes were already detected to influence IBD progression in different ways. Exosomes secreted from platelets were also found (35, 36).

### Exosomes From Physiological Fluids

Intestinal luminal exosomes may contribute to the diagnosis of IBD by isolating corresponding internal mRNAs inside, and this may become a popular clinical method to diagnose IBD in the future (37). Exosomes from intestinal lymph containing high complement C3/C5 and albumin are studied (38–40). Since complement C3/C5 is involved in the initiation of inflammatory responses, exosomal C3/C5 from intestinal lymph may also indicate the potential for IBD diagnosis and treatment.

### Exosomes From Plants

Some edible plants like *Curcuma longa* and grapes may provide exosomes, contributing to IBD alleviation. The medical herb *Curcuma longa*-derived exosomes may potentially inactivate nuclear factor-kappaB (NF-κB) pathway to ameliorate colitis and promote intestinal wound repair (41). Oral administration of exosome-like nanoparticles from grape juice protects mice against DSS-induced colitis (42).

## Effects of Exosomes/Exosomal Components on IBD

Exosomes contain various biological components such as nucleic acids, proteins, and other components like lipids, chemical drugs, and natural substances et al. These nucleic acids, proteins, and other components can be transported by exosomes and not only facilitate or alleviate the pathological processes, but also play a role in the diagnosis and therapy of IBD (7, 43, 44).

Exosomal components may be divided into two types according to whether their effects on IBD are dependent on the exosome structure itself. One type of exosomal component plays a role in IBD and is dependent on the exosome as a vehicle or for exosome-mediated transport. Peptide-MHC II complexes are one kind of important mediator in the communication between IECs and DCs, and the communication is dependent on exosome loaded with peptide-MHC II complexes (45). The exosomal pathway can significantly increase the capacity of antigen presenting of IECs. Although exosome-induced regulation in IBD mainly depends on the internal biological components, exosome structure itself is also very important. Exosome structure-induced transport between different tissues, even across different species, helps biological components to be involved in the regulation of IBD. The structure of exosome membrane can affect the functions, especially the kinetics of some components inside. It has been confirmed that the biological components inside exosomes show more stability than those with no exosome loading. In a colitis model, TGF-β1 in exosomes is more stable than free TGF-β1 cytokine when digested by trypsin, and disruption of exosome structure can decrease the efficacy of TGF-β1 (20).

The other type of exosomal components can also affect IBD progression but the effects are not dependent on the exosome structure. In other words, these types of components are widely distributed throughout the human body, are involved in IBD progression and can be detected in the exosome. GRP78, an endoplasmic reticulum stress marker protein, is involved in IBD. GRP78 is widely expressed in intestine, and it can also be loaded in exosomes in IECs (46, 47). The effects of the exosome on this type of component-induced regulation of IBD need to be deeply studied.

Some exosomes/exosomal components have been proved to influence IBD, and these exosomes are shown in Table 1.

**Table 1:** Effects of exosomes/exosomal components on IBD.

**Exosomal components**	**Origins**	**Effects and mechanisms**	**Significance**	**Exosome structure effects**	**References**
MiR21	Substance P-induced colon ECs	Regulates immune cell proliferation and migration	Treatment	Membrane carrier	(48)
ANXA1	Serum of DSS induced acute UC mice	Anti-inflammation, protection of epithelial barrier	Treatment	Enhancement of ANXA1 efficacy	(49)
PSMA7	Saliva of IBD patients	High expression, be found only expressed in IBD	Diagnosis	Protection and long-distance transportation	(17)
TKT, TLN1, WDR1, NUCB2, BASP1, PSMB7, IGHV4OR	Saliva of IBD patients	Be found only expressed in IBD	Diagnosis	Membrane carrier	(17)
IBD acute phase proteins and Ig	Serum of acute colitis mice	Induces MAPK and TNF-α activation, indicates macrophage activation	Diagnosis/Treatment	Membrane carrier	(16)
Dietary proteins (immunogens)	IECs	Promotes antigen presentation, increases intestinal permeability	Treatment	Transfer the components	(50)
TGF-β1	TGF-β1 gene-modified DCs	Inhibits Th17 cell development	Treatment	Enhances the effects of TGF-β1	(20)
Phenolic compounds	Grape juice	Inhibits TNF-α and NF-κB	Treatment	Drug carrier	(14, 51)

*ANXA1/2, Annexin A1/2; BASP1, brain acid soluble protein 1; CD, crohn's disease; DC, dendritic cell; DSS, dextran sulfate sodium; EC, epithelial cell; IEC, intestinal epithelial cell; IBD, inflammatory bowel disease; IGHV4OR, putative V-set and immunoglobulin (Ig) domain-containing-like protein; miRNA, microRNA; miR21, miRNA21; NF-κB, nuclear factor-kappaB; NUCB2, nucleobindin; PSMA7, proteasome subunit alpha type-7; PSMB7, proteasome subunit beta type-7; TKT, transketolase; TLN1, talin-1; TGF-β1, transforming growth factor-beta1; TNF, tumor necrosis factor; UC, ulcerative colitis; WDR1, WD repeat-containing protein 1*.

MiRNAs are small non-coding RNAs mediating post-transcriptional gene regulation by binding to the 3′-untranslated region or opening reading frame region of target mRNA to induce gene silencing (52). MiRNA21 (miR21) has been found to bind to specific mRNAs to regulate protein production or cause RNA cleavage. Exosomes from substance P-induced colonic ECs carried with miR21 exacerbate colitis through stimulating immune cell proliferation and migration (48). It may be an alternative pathway to block substance P-induced exosome secretion in the treatment of IBD.

ANXA1, an endogenous protein expressed in responsive cells like phagocytes or epithelial cells, can promote inflammation resolution. Exosomes carried with ANXA1 can be detected in the serum of DSS induced acute UC mice (49). These exosomes are proven to facilitate anti-inflammation and recovery of epithelial barrier function in IBD, which contributes greatly to IBD treatment. For the diagnosis of IBD, PSMA7 released from salivary exosomes provide a more convenient way since it was found to be expressed much higher in patients with IBD (both in CD and UC), which indicates it to be an ideal biomarker for IBD diagnosis (17).

TGF-β1 is one kind of major cytokine with a prominent immunosuppressive effect, and TGF-β1 in exosomes is believed to have therapeutic potential in IBD. TGF-β1 gene-modified DC-produced exosomes can inhibit the development of IBD by inhibiting Th17 (20). Besides, these membrane exosome structures were also proven to enhance the effects of TGF-β1 inside, which means that exosome structure can improve the therapeutic potential of TGF-β1 in IBD.

The natural substance phenolic compounds are also found to be packaged in exosomes (14, 51). The exosomes loaded with phenolic compounds are isolated from grape juice, which can inhibit colitis inflammation by decreasing TNF-α and NF-κB. Plants or food-originated exosomes loaded with immunoregulatory agents may be another novel choice in IBD therapy, because higher efficacy of exosomes containing chemotherapeutic drugs compared with free drugs is found (53).

## Exosomes/Exosomal Components Which Have Potential Effects on IBD

In IBD development, many substances including RNAs, proteins and other substances have been involved. However, it is necessary to compare the effects of the free substances with exosomes loaded with the substances. Some exosomes/exosomal components may have potential functions in IBD progression, which may be valuable to be studied in the future as potential targets for IBD diagnosis and treatment. These exosome/exosomal components are shown in Supplementary Table 2.

### Exosomal Nucleic Acids

Long non-coding RNA (LncRNA) can bind to miRNAs and interact with various RNA molecules. Furthermore, changes in LncRNAs are related to IBD (54, 55). Exosomal LncRNA growth arrest-specific 5 has been detected in IBD and its functional course might be related to miR21, as increased miR21 expression has been confirmed to lead to IBD (48, 54, 55).

Exosomal miRNAs have been proven to be very useful as biomarkers, based on a study using clinical samples from IBD patients. These functional exosomal miRNAs may not only influence immunity, but also influence the intestinal barrier function in IBD. The ways in which exosomal miRNAs affect IBD are introduced below.

#### Exosomal miRNAs Regulates Immunity

IBD is the consequence of a dysregulated mucosal immune system, which has been extensively studied. Exosome-mediated transfer of miRNAs is common among immune cells, contributing to the coordination of immune system in IBD (14). Exosomal miRNAs regulate immunity via multiple pathways including modulating functions of DCs, T cells, and macrophages, as well as NF-κB related signaling pathways. Targeting exosomal miRNAs signaling pathways to modulate immune dysfunction may become a promising direction for IBD treatment.

Exosomal miRNAs derived from DC exosomes might interact with DCs or recipient DCs. For example, two critical miRNAs, miR-155, and miR-146a are believed to modulate inflammation and enter into recipient DCs to mediate target gene repression (56). Deficiency of miR-155 has been demonstrated to ameliorate intestinal inflammation by suppressing the development and function of CD103^+^CD11b^+^DCs in the lamina propria of the intestine (27). T cells are also activated by miR-1246 through activating the proinflammatory nuclear factor in active IBD (57, 58).

Some exosomal miRNAs promote an inflammatory reaction by regulating the NF-κB signaling pathway, and the activation of this pathway is induced in UC, which promotes the transcription of various pro-inflammatory cytokine genes (59). The increased expression of miR-16 has been shown to inhibit adenosine A2a receptor expression to mediate the NF-κB signaling pathway, and thus, influence the immune and inflammatory responses in UC (59).

#### Exosomal miRNAs Regulate Intestinal Barrier Function

There is growing evidence that intestinal barrier dysfunction is an important pathogenic mechanism in IBD (60). Different macrophage subtypes like lamina propria monocytes and M1 macrophages decrease tight junction (TJ) proteins, leading to TJ barrier dysfunction in IBD (61). Deficiency of miR-155 has been proven to ameliorate IBD inflammation in colitis mice (27). Krüppel-like factor 5-over-expressing vascular smooth muscle cell-derived exosomes loaded with the critical miR-155 (proved to ameliorate IBD inflammation) can be transported into other cells, like vascular endothelial cells, to destroy the integral endothelium (28). However, it remains elusive whether exosomes loaded with miR-155 have regulatory effects on intestinal barrier function in IBD. Conversely, miR-146b derived from DC exosomes has the ability to activate NF-κB to improve epithelial barrier function (56, 62).

One of the miRNAs derived from exosomes can regulate both immune cells and intestinal barrier function in IBD. MiR-223 from thrombin-activated platelet-derived exosomes can block the MAPK pathway (down regulating the phosphorylation of p38, c-JNK, and ERK) and the NF-κB p65 nuclear translocation to inhibit intercellular adhesion molecule-1 in the endothelial cell inflammation (63, 64). More studies are needed to study the effects of these miRNAs packaged by exosomes on IBD progression.

### Exosomal Proteins

Potential effects of exosomal proteins on IBD are of great value. Exosomal proteins can not only affect the occurrence and development of diseases, but can also be used as indicators of disease status (12, 44).

#### Exosomal Proteins Regulate Immunity

During the progression of IBD, the cytokine profiles and expression of T cells can be regulated by exosomal proteins. Exosomal protein human leukocyte antigen-G (HLA-G) is a MHC class I antigen that can mediate apoptosis of CD8^+^T cells through regulating the activity of natural killer cells and DCs. HLA-G might mediate inflammation tolerance, and normalization of HLA-G may contribute to IBD therapy (29, 30). Further studies are required to determine whether the same signaling pathway is involved in both exosomal HLA-G and free HLA-G-induced regulation of colitis.

#### Exosomal Proteins Regulate Intestinal Barrier Function

Cellular prion protein (PrPC), Annexin A1 (ANXA1) and glucose-regulated protein 78 (GRP78) have been found to be packaged in exosomes. PrPC is mostly found in platelets and can be released from platelet-derived exosomes (35). ANXA1, the potent endogenous pro-resolving mediator, is released from IECs into the extracellular space (49). GRP78 can be released from colon cancer cells via exosomes and participate in cell-cell communication (46). PrPC was found to maintain TJ barrier function and protect lateral junctional complexes in UC and CD (36). The ANXA1 mimetic peptide, Ac2-26, delivered from polymeric NPs, accelerated the recovery of epithelial barrier function (49). Expression of the endoplasmic reticulum stress marker protein, GRP78, has been found to be decreased in IBD, and the effects of GRP78 is to maintain the function and structure of the colon epithelium (47).

#### Exosomal Proteins Regulate Intestinal Microbiota

Dysbiosis of intestinal microbiota, including abnormal immune responses, infections, dietary habits, and administration of antibiotics is an important factor in the occurrence and development of IBD (65). DC-derived exosomal heat shock protein 73 (HSP73) and IEC-derived exosomal HSP72 have already been identified in numerous studies (66, 67). As potent adjuvant for eliciting immune responses, HSP70 is known to induce proinflammatory cytokine production via the myeloid differentiation factor 88/NF-κB signal transduction pathway. It can utilize receptors for both Gram-positive (TLR2) and negative bacteria (TLR4) to stimulate the proinflammatory signal in a CD14-dependent fashion (67).

### Other Exosomal Components (Mainly Lipids) in IBD

Lipid rafts are sphingolipid/cholesterol-enriched domains of the exosomal membrane (68). Lipid raft disruption occurring prior to increased intestinal permeability may result in epithelial barrier dysfunction (69). Research into exosomal cholesterol in IBD of course cannot be ignored, especially in UC, as it has been shown that cholesterol might be useful in the diagnosis or evaluation of this disease. It has previously been demonstrated that cholesterol in the feces of UC patients is increased (68, 70), and that it can inhibit pyroptosis signaling to ameliorate experimental colitis (70). Furthermore, a decrease in high-density lipoprotein-cholesterol in IBD patients has been reported (71).

Plant-origin glucosylceramide (a cellular lipid) can suppress colon inflammation in IBD (72), and α-galactosylceramide can bind with CD1 to inhibit activation of natural killer T cells in the intestinal epithelium (73). These findings may indicate the potential effects of exosomal glucosylceramide on IBD.

One study demonstrated that exosomes derived from enterobacterias could stimulate the intestinal epithelium to produce exosome-like NPs containing sphingosine-1-phosphate, C-C motif chemokine 20, and prostaglandin. These contents could increase Th17 cell proliferation through the myeloid differentiation factor 88-mediated pathway and cause intestinal inflammation (74).

Glycoconjugates on the surface of bovine milk exosomes could stimulate uptake of exosomes, especially in human colon Caco-2 cells (75). These exosomal glycoconjugates may replicate the function of host exosomes and provide novel ways to treat IBD.

## Summary

Exosomes have involved significant potential in the last few decades because of their novel roles in multiple biological signaling pathways. The functions and detailed mechanisms of exosomes in the IBD process remains understudied (Figure 1), however, more and more results suggest the significant interest of exosomes in diagnosis and treatment of IBD with both opportunities and challenges.

Firstly, the development of novel and non-invasive diagnostic methods is of great importance. The advances in exosome-related researches suggest potential significance of exosomes in IBD diagnosis. Studies showed that exosomes isolated from serum or saliva in IBD patients can be used as a biomarker for clinical diagnosis (16, 17). One obstacle for the application of exosomes in IBD diagnosis is the discrepancy between the sensitivity and specificity of circulating RNAs and/or proteins in diagnosing various intestinal inflammation related diseases. To find reliable and specific exosomal RNAs and/or proteins may be beneficial for the widespread application of exosomes in the diagnosis of IBD.

Secondly, exosomes induced “long-distance” cell-cell communication in IBD and underlying mechanisms may suggest some novel opinions to uncover the pathological mechanisms of IBD, even for other diseases which has been confirmed to be related to intestinal dysfunctions, like Alzheimer disease, Parkinson's disease, and cardiovascular diseases (76–78). Exosomes from immune cells, especially DCs, can regulate immunostimulation or immunosuppression in IBD immunity (19). IECs can also secrete exosomes inducing immunity tolerance, and tend to be regulated by exosomes from IECs or even immune cells when IBD occurs (14, 21). These communications are mainly completed by exosomal components (RNAs, proteins, or lipids) inside, and finally lead to the regulation of intestinal homeostasis. Actually in IBD, whether inflamed intestine-derived exosomes transport regulators from intestine to other tissues to exert regulatory effects still remains unknown. In other words, to study exosome-induced cell-cell communication between intestine and other tissues like brain and cardiovascular tissues is of interest.

Existing drugs for the treatment of IBD still have significant limitations, especially for moderating severe UC, or CD (79). Thus, both sequencing new drugs and optimizing pharmaceutical dosage form are needed. Last but not least, the use of exosome containing biological contents/chemical contents as drug delivery vehicles is of considerable interest due to their good biodistribution and inherent biocompatibility. The exosomes isolated from medical herb plants, immune cells, and other cells *in vitro* could alleviate marine colitis. Exosome-induced regulation in IBD mainly depends on internal biological components, and the structure of exosome can affect the functions, especially the metabolic dynamics of the components inside. More studies are needed to compare the effects of exosome-loaded drug with the free drug without exosomes loaded. In addition, it is believed that designing new drug dosage form using exosome-like-structure may provide new insights into IBD treatment. Both animal and clinical studies are required to investigate the efficacy of exosomes on IBD before it can be used.

## Author Contributions

DC drafted and edited the manuscript. DC, HZ, LW, CL, YYu, YYi, and JW edited the manuscript. DC, HZ, LW, and CL drafted and edited the figures and figure legends. All authors approved the work for publication.

### Conflict of Interest Statement

The authors declare that the research was conducted in the absence of any commercial or financial relationships that could be construed as a potential conflict of interest.
