# Supplementary material for: Exosome-Induced Regulation in Inflammatory Bowel Disease
Source: Front Immunol. 2019 Jun 28;10:1464. doi: 10.3389/fimmu.2019.01464 (PMC6611439; doi:10.3389/fimmu.2019.01464)
Supplement: Supplementary file 1 [file Data_Sheet_1.PDF]

Supplementary Table 1. Various origins of exosomes

Origins of exosomes	Effects/Mechanisms	Reference
**DCs**		
TGF-β-gene-modified DCs	Alleviation of DSS-induced colitis through increasing Treg cells	[1]
IL-10-treated DCs	Inhibition of trinitrobenzene sulfonic acid-induced colitis by up regulating IL-10 mRNA and promoting Tregs expansion	[2]
SEA-treated immature bone marrow-derived DCs	Alleviation of DSS-induced colitis through immunosupression	[3]
CD103+CD11b+ DCs	Have the potential to the alleviation of IBD by inhibiting miR155 expression inside and suppressing the function of CD103+CD11b+DCs	[4; 5; 6]
**Exosomes from other cells**		
IECs	Modulation of IBD conditions through expressing high levels of MHC I and II molecules in inflammation associated with MHC II-dependent antigen-specific tolerance<br>Exosomes from IEC-6 have the potential to aggravate IBD by down regulating c-Myb and Bcl-2 expression and thus inducing colonic EC apoptosis in active IBD through miR150 inside	[7; 8; 9]
Human umbilical cord mesenchymal stem cells	Alleviation of DSS-induced colitis through reducing IL-7 expression in macrophages	[10]
G-MDSC	Alleviation of DSS-induced colitis through inhibiting proliferation	[11]

	of Th1 cells as well as CD4+T cells, and promoting Treg cell expansion	
ET-BSP	Stimulation of intestinal epithelium and production of intestinal mucosa-derived exosome-like NPs by modulating CCL20, prostaglandin E2-required proliferation of Th17 cells, and MyD88-mediated pathway which may contribute to the development of gut inflammation	[12]
Caco-2 cells infected with rotavirus or human intestinal epithelial T84 cells/THP-1 macrophages infected with CD-associated adherent-invasive escherichia coli	These exosomes secreted from special cells have the possibility to affect IBD conditions	[13; 14]
**Exosomes from physiological fluids**		
Intestinal luminal fluid	Diagnosis and treatment of IBD	[15]
Saliva	Proteasome subunit alpha type 7 released from salivary exosomes provide an accurate way to diagnose IBD since it was found to express much higher in patients with IBD, both in CD and UC	[16]
Circulating serum	Modulation of macrophage activation, which is essential for the maintenance of intestinal homeostasis, plays a key role in the pathogenesis of IBD	[17]

Milk	Influences IEC immunity to modulate permeability of intestinal barrier	[18; 19]
Intestinal lymph	Have the potential to accelerate IBD through their contents such as complement C5, fibronectin, plasminogen and so on	[20; 21; 22]
**Exosomes from edible plants**		
Curcuma Longa	Alleviation of colitis through inhibiting NF-κB pathway	[23]
Grapes	Penetrates the intestinal mucus barrier and induces $Lgr5^{hi}$ intestinal stem cells through the Wnt/testinnin pathway to alleviate IBD	[24]

Note: CCL20, C-C motif chemokine 20; CD, Crohn's disease; DC, dendritic cell; DSS, dextran sulfate sodium; ET-BSP, enterobacteria; G-MDSC, granulocytic myeloid-derived suppressor cell; IBD, inflammatory bowel disease; IEC, intestinal epithelial cell; IL, interleukin; mRNA, messenger RNA; MHC, major histocompatibility complex; MyD88, myeloid differentiation factor 88; NPs, nanoparticles; NF-κB, nuclear factor-kappaB; PSMA7, proteasome subunit alpha type 7; SEA, S.japonicum soluble egg antigen; TGF, transforming growth factor; Treg, regulatory T cell; UC, ulcerative colitis

Refferences

[1] Z. Cai, W. Zhang, F. Yang, L. Yu, Z. Yu, J.H. Pan, L. Wang, X. Cao, and J. Wang, Immunosuppressive exosomes from TGF-β1 gene-modified dendritic cells attenuate Th17-mediated inflammatory autoimmune disease by inducing regulatory T cells. Cell Research 22 (2012) 607.

[2] X. Yang, S. Meng, H. Jiang, T. Chen, and W. Wu, Exosomes derived from interleukin-10-treated dendritic cells can inhibit trinitrobenzene sulfonic acid-induced rat colitis. Scandinavian journal of gastroenterology 45 (2010) 1168-77.

[3] L. Wang, Z. Yu, S. Wan, F. Wu, W. Chen, B. Zhang, D. Lin, J. Liu, H. Xie, and X. Sun, Exosomes Derived from Dendritic Cells Treated with Schistosoma japonicum Soluble Egg Antigen Attenuate DSS-Induced Colitis. Frontiers in Pharmacology 8 (2017) 651.

[4] C. Wang, C. Zhang, L. Liu, A. Xi, B. Chen, Y. Li, and D. Jie, Macrophage-Derived mir-155-Containing Exosomes Suppress Fibroblast Proliferation and Promote Fibroblast Inflammation during Cardiac Injury. Molecular Therapy the Journal of the American Society of Gene Therapy 25

(2017) 192.

[5] Y. Xu, B. Zhang, X. Zhu, C. Li, W. Song, and H. Tang, miR155-deficient alleviated intestinal inflammation by downregualting the development and function of CD103+CD11b+DC in the lamina propria of intestine. The Journal of Immunology 198 (2017) 62.8-62.8.

[6] B. Zheng, W.N. Yin, T. Suzuki, X.H. Zhang, Z. Yu, L.L. Song, L.S. Jin, Z. Hong, Z. Hong, and J.S. Li, Exosome-Mediated miR-155 Transfer from Smooth Muscle Cells to Endothelial Cells Induces Endothelial Injury and Promotes Atherosclerosis. Molecular Therapy the Journal of the American Society of Gene Therapy 25 (2017) 1279.

[7] K. Bakirtzi, D. Iliopoulos, and C. Pothoulakis, 1093 Identification and Characterization of Colonic-Exosomes: micro-RNA Mediated Horizontal Gene Transfer Within the Colonic Epithelia In Vitro and In Vivo. Gastroenterology 150 (2016) S218-S218.

[8] W. Sakamoto, T. Masuno, H. Yokota, and T. Takizawa, Expression profiles and circulation dynamics of rat mesenteric lymph microRNAs. Molecular Medicine Reports 15 (2017) 1989-1996.

[9] Z. Bian, L. Li, J. Cui, H. Zhang, Y. Liu, C.Y. Zhang, and Z. Ke, Role of miR-150-targeting c-Myb in colonic epithelial disruption during dextran sulphate sodium-induced murine experimental colitis and human ulcerative colitis. Journal of Pathology 225 (2011) 544-553.

[10] F. Mao, Y. Wu, X. Tang, J. Kang, B. Zhang, Y. Yan, H. Qian, X. Zhang, and W. Xu, Exosomes Derived from Human Umbilical Cord Mesenchymal Stem Cells Relieve Inflammatory Bowel Disease in Mice. BioMed research international 2017 (2017) 5356760.

[11] Y. Wang, J. Tian, X. Tang, K. Rui, X. Tian, J. Ma, B. Ma, H. Xu, L. Lu, and S. Wang, Exosomes released by granulocytic myeloid-derived suppressor cells attenuate DSS-induced colitis in mice. Oncotarget 7 (2016) 15356-68.

[12] Z. Deng, J. Mu, M. Tseng, B. Wattenberg, X. Zhuang, N.K. Egilmez, Q. Wang, L. Zhang, J. Norris, and H. Guo, Enterobacteria-secreted particles induce production of exosome-like S1P-containing particles by intestinal epithelium to drive Th17-mediated tumorigenesis. Nature Communications 6 (2015) 6956.

[13] D. Bautista, L.S. Rodríguez, M.A. Franco, J. Angel, and A. Barreto, Caco-2 cells infected with rotavirus release extracellular vesicles that express markers of apoptotic bodies and exosomes. Cell Stress & Chaperones 20 (2015) 1-12.

[14] J. Carrière, A. Bretin, A. Darfeuillemichaud, N. Barnich, and H.T. Nguyen, Exosomes Released from Cells Infected with Crohn's Disease-associated Adherent-Invasive Escherichia coli Activate Host Innate Immune Responses and Enhance Bacterial Intracellular Replication. Inflammatory Bowel Diseases 22 (2016) 516-528.

[15] S. Mitsuhashi, L. Feldbrugge, E. Csizmadia, M. Mitsuhashi, S.C. Robson, and A.C. Moss, Luminal Extracellular Vesicles (EVs) in Inflammatory Bowel Disease (IBD) Exhibit Proinflammatory Effects on Epithelial Cells and Macrophages. Inflammatory bowel diseases 22 (2016) 1587-95.

[16] X. Zheng, F. Chen, Q. Zhang, Y. Liu, P. You, S. Sun, J. Lin, and N. Chen, Salivary exosomal PSMA7: a promising biomarker of inflammatory bowel disease. Protein Cell 8 (2017) 686-695.

[17] W.Y. Wong, M.M. Lee, B.D. Chan, R.K. Kam, G. Zhang, A.P. Lu, and W.C. Tai, Proteomic profiling of dextran sulfate sodium induced acute ulcerative colitis mice serum exosomes and their immunomodulatory impact on macrophages. Proteomics 16 (2016) 1131-45.

[18] H. Izumi, M. Tsuda, Y. Sato, N. Kosaka, T. Ochiya, H. Iwamoto, K. Namba, and Y. Takeda, Bovine milk exosomes contain microRNA and mRNA and are taken up by human macrophages. Journal of Dairy Science 98 (2015) 2920-33.

[19] B. Lönnerdal, X. Du, Y. Liao, and J. Li, Human milk exosomes resist digestion in vitro and are internalized by human intestinal cells. Faseb Journal 29 (1 supplement) (2015) 121.3.

[20] N. De Silva, M. Samblas, J.A. Martinez, and F.I. Milagro, Effects of exosomes from LPS-activated macrophages on adipocyte gene expression, differentiation, and insulin-dependent glucose uptake. Journal of physiology and biochemistry 74 (2018) 559-568.

[21] J. Caradec, G. Kharmate, E. Hosseini-Beheshti, H. Adomat, M. Gleave, and E. Guns, Reproducibility and efficiency of serum-derived exosome extraction methods. Clinical biochemistry 47 (2014) 1286-92.

[22] H. Ryu, E. Gounaris, M. Kron, C. Waltenbaugh, Z.J. Zhang, and T.A. Barrett, Novel Pathway for TNF-Mediated Albumin Losses in IBD. Gastroenterology 140 (2011) S-502-S-502.

[23] M. Zhang, and D. Merlin, Curcuma Longa-Derived Nanoparticles Reduce Colitis and Promote Intestinal Wound Repair by Inactivating the NF-κB Pathway. Gastroenterology 152 (2017) S567.

[24] S. Ju, J. Mu, T. Dokland, X. Zhuang, Q. Wang, H. Jiang, X. Xiang, Z.B. Deng, B. Wang, L. Zhang, M. Roth, R. Welti, J. Mobley, Y. Jun, D. Miller, and H.G. Zhang, Grape exosome-like nanoparticles induce intestinal stem cells and protect mice from DSS-induced colitis. Molecular therapy : the journal of the American Society of Gene Therapy 21 (2013) 1345-57.
