# Supplementary material for: Exosome-Induced Regulation in Inflammatory Bowel Disease
Source: Front Immunol. 2019 Jun 28;10:1464. doi: 10.3389/fimmu.2019.01464 (PMC6611439; doi:10.3389/fimmu.2019.01464)
Supplement: Supplementary file 2 [file Data_Sheet_2.PDF]

Supplementary material Table 2. Mechanisms of exosomes/exosomal components which have potential effects on IBD.

Components	Function	Origins	Effects/Mechanisms	Significance	Functions of exosome structure	Reference
**Exosomal lncRNAs**						
LncRNA GAS5	Long, non-coding RNA up regulates glucocorticoid in-resistant cells	—	Promotes apoptosis of macrophages or inhibits glucocorticords activity	Treatment	Membrane carrier of high level lncRNA GAS5	[1; 2]
**Exosomal miRNAs**						
MiR155	Short RNA molecules binding to specific mRNAs to regulate protein production or cause RNA cleavage; Stimulates endothelium barrier dysfunction and intestinal inflammation in IBD	Macrophages, DCs, KLF5-overex pressing VSMCs	Deficiency of exosomal miR155 may ameliorate intestinal inflammation by suppressing the development and function of CD103+CD11b+DC in the lamina propria of intestine in IBD; KLF5-overexpressing VSMCs-derived exosomes loaded with miRNA155 can transport into ECs to modulate endothelium barrier dysfunction through downregulation of TJ protein expression and disruption of this process may alleviate IBD	Treatment	As a paracrine messenger to mediate the cell-cell communication	[3; 4; 5]
MiR146b	Up regulates NF-κB to accelerate IBD inflammation	DCs	Aggravates intestinal inflammation by up regulating NF-κB and deficiency of it may ameliorate IBD course	Treatment	Membrane carrier	[6; 7]
MiR1246	Activates macrophages and T cells to aggravate IBD inflammation	Detected in sera	Suppresses the anti-inflammation by reprogramming macrophages or activates the proinflammatory nuclear factor of activated T cells, and may contribute to the effective indication as a biomarker of active IBD	Treatment/ Diagnosis	Drives macrophage reprogramming	[8; 9]
MiR16	Activates NF-κB signaling pathway to accelerate UC	Detected in human milk	Inhibition of adenosine A2a receptor expression to activate NF-κB signaling pathway in UC	Treatment	Able to survive digestion and being taken up by enterocytes	[10; 11]
MiR29b	Up regulates in active UC	Detected in bovine milk	Accumulated and transported by colon cells in IBD; Up regulates in active UC	Treatment	Able to survive digestion and being accumulated and	[12; 13]

					transported by intestinal mucosal cells to peripheral tissues	
MiR200c	Up regulates in IBD	Detected in bovine milk	Up regulates and affects gasotransmitter pathways in IBD	Treatment	Able to survive digestion and being transported by intestinal mucosal cells to across the basolateral membrane for subsequent delivery to peripheral tissues	[12; 14]
MiR223	Activates NLRP3 inflammasome and facilitates the release of IL-1β and IL-18 to alleviate colitis	Thrombin-activated platelets	Down regulates MAPK and NF-κB pathway to inhibit ICAM-1 expression in inflammation; Exosomal miR223 may regulate the activation of the NLRP3 inflammasome and the release of IL-1β and IL-18 to attenuate early colitis	Treatment	Membrane carrier	[15; 16]
MiR22	Up regulates in CD	Detected in human milk	Stimulates CD progress	Diagnosis	Able to survive digestion and being taken up by enterocytes	[13; 17]
MiR375	Up regulates in active CD	Detected in serum	Stimulates active CD progress	Diagnosis	Provides stability for cell-free miR375 in various bodily fluids	[13; 18]
MiR150	Down regulates c-Myb and Bcl-2 to induce colonic EC apoptosis in active IBD	IEC-6	Down regulates c-Myb and Bcl-2 expression to induce colonic EC apoptosis in active IBD and deficiency of it may contribute to IBD therapy	Treatment	—	[19; 20]
**Exosomal proteins**						
ANXA2	A protein facilitates phagocytosis of apoptotic ECs	—	Exosomal Annexin A2 activation enhances phagocytosis of apoptotic ECs, while its inhibition may prevent membrane-anchored TNF-α shedding in IBD	Treatment	Membrane carrier	[21; 22]
Ubiquitinated HLA-G	Secreted soluble molecules belonging to the non-classical HLA class I family; Expresses only on	HLA-G1-transfected cells	Modulates immune-regulatory tolerance, the secretion of IL-10 in response to inflammation and the mucosal immunity in UC	Treatment	Membrane carrier	[23; 24]

	maternal–fetal interface and immune privileged adult tissues					
S100A8	A pro-inflammatory protein increased in serum and mucosa of IBD children	MDSC	Potential new biomarker in IBD diagnosis or regulates T cell responses	Treatment/ Diagnosis	Membrane carrier	[25; 26]
S100A9	A pro-inflammatory protein increased in serum and mucosa of IBD children	MDSC	Facilitates UC by activating STAT3 or T cell responses and down regulation of it may contribute to IBD therapy	Treatment	Membrane carrier	[25; 26]
TG2	A functional enzyme increased in IBD	TG2 proficient cells	Inhibition of exosomal TG2 may contribute to IBD treatment	Treatment	Membrane carrier	[27; 28]
Complement C3	An inflammatory protein	Intestinal lymph	Potential autoantigen in IBD	Diagnosis	Membrane carrier	[29; 30; 31]
Complement C5	Aggravates colitis by up regulating the mucosal expression of TNF-α, IL-1β and IL-6	Intestinal lymph	Inhibition of exosomal complement component C5 may ameliorate colitis by reducing the mucosal expression of TNF-α, IL-1β and IL-6	Treatment	Membrane carrier	[29; 30; 31]
Fibronectin	Induces colitis	Intestinal lymph	Induces colitis mediated by integrin-linked kinase	Diagnosis	Membrane carrier	[32; 33]
Plasminogen	Increased in UC	Intestinal lymph	Indicates clinical activity of UC	Diagnosis	Membrane carrier	[34; 35]
Thymic stromal lymphopoietin	Products of harmless antigens	IEC	Involves in the conditioning of DCs in IBD	Treatment	Membrane carrier	[36]
Integrin αvβ6	Involving in the tolerogenic DC conditioning.	IEC	Increased expression of TGF-β in DC conditioning and Treg cell induction in IBD	Treatment	Membrane carrier	[36]
Beta-defensin 2	Anti-microbial peptides	IEC apical side	Exosomes from IEC apical side may be stimulated by protozoan parasite Cryptosporidium parvum to produce and secret in the luminal through the activation of TLR4 signaling	Treatment	Membrane carrier	[36]
Cathelicidin-37	Anti-microbial peptides	IEC apical side	Exosomes from IEC apical side may be stimulated by protozoan parasite Cryptosporidium parvum to produce and secret in the luminal through the activation of TLR4 signaling	Treatment	Membrane carrier	[36]
GRP78	As an endoplasmic reticulum stress marker	Unclear	Maintains normal colonic epithelium function in IBD	Treatment	As a paracrine messenger to mediate	[37; 38]

	protein to maintain normal colonic epithelium function in IBD				the cell-cell communication, or as a biomarker of disease	
PrPC	A glycosylphosphatidylinositol-anchored glycoprotein located in cell–cell junctions and interacts with desmosome proteins in the intestinal epithelium	Platelet	Regulates TJ barrier function in both UC and CD	Treatment	Membrane carrier	[39; 40]
Peroxisome proliferator-activated receptor-γ	A nuclear receptor regulates differentiation and proliferation of adipocyte, and functions of immune cells as well as inflammatory cells	Human plasma	Contributes to the paracrine transfer of nuclear receptors in IBD, especially UC and down regulation of it may contribute to IBD therapy	Treatment	Membrane carrier	[41; 42]
Albumin	Mediated by TNF to induce IBD	Intestinal lymph	Mediated by TNF to induce IBD and downregulation of it may contribute to IBD therapy	Treatment	Membrane carrier	[29; 30; 31]
HSP70 protein family (including HSP72 and HSP73)	Regulates lymphocyte activity an d cytokine production	—	Exosomal HSP70 protein may regulate lymphocyte activity and cytokine production; HSP72 may regulate intestinal microbiota profile	Diagnosis/ Treatment	—	[43; 44]
**Special contents**						
Sphingosine-1-phosphate, CCL20, prostaglandin	CCL20 (cytokine) and prostaglandin (pro-inflammatory mediator) involve in the recruitment and proliferation of Th17 cells; Sphingosine-1-phosphate((human sequence-specific DNA binding protein) stimulates the production of CCL20+PGE2+S1P+	Intestinal mucosa	Stimulated by ET-BSPs exosomes, may modulate E2-required proliferation of Th17 cells through the MyD88-mediated pathway and cause intestinal inflammation in IBD	Treatment	As a membrane carrier to involve in intracellular communication across species	[45]

	exosomes					
Cholesterol	A kind of lipid suppresses IBD inflammation or even inhibits pyroptosis signaling to alleviate IBD	—	Inhibits pyroptosis signaling to ameliorate experimental colitis	Treatment	Membrane carrier	[46; 47]
Glucosylceramide	A kind of lipid suppresses IBD inflammation	—	Suppresses colon inflammation in IBD or bind with CD1 to inhibit activation of NK T cells in intestinal epithelium	Treatment	Membrane carrier	[48; 49]
Lysophosphatic acid	A kind of lipid activates proinflammatory mediators of IBD	—	Activates proinflammatory mediators in the arachidonic pathway in chronic colitis and down regulation of it may contribute to IBD therapy	Treatment	Membrane carrier	[46; 50]
Phosphatidylcholine	A kind of lipid induces inflammation in UC	—	Inhibitory effects of corticosteroid in patients with chronic steroid-refractory UC, or induce inflammation in UC and down regulation of it may contribute to IBD therapy	Treatment	Membrane carrier	[46; 51]
Sphingomyelin	A kind of lipid alleviates inflammation	Mainly from B-lymphocyte	Modulates inflammation by activating peroxisome proliferator-activated receptor-γ, and decreased exosomal sphingomyelin of it may activate cathepsin D as a mediator of apoptosis related to disruption of epithelial barrier in IBD	Treatment	Membrane carrier	[46; 52]
Phosphatidylinositol	A kind of lipid	Mainly from B-lymphocytes, mast cells and DCs	Deficiency of exosomal phosphatidylinositol may protect against endoplasmic reticulum stress in IBD	Treatment	Membrane carrier	[46; 53]
Curcumin	A polyphenol derived from the herbal remedy and dietary spice turmeric	Detected in milk	Alleviation of IBD	Treatment	Provides stability in PBS; Survives digestion and being easier to cross the intestinal barrier	[54]

Note: ANXA2, Annexin A2; ApoA-I, apolipoprotein A-I; CCL20, C-C motif chemokine 20; CD, crohn's disease; circRNAs, circular RNAs; CLDN8, claudin-8; DC, dendritic cell; DSS, dextran sulfate sodium; EC, epithelial cell; EGFR, epidermal growth factor receptor; ET-BSP, enterobacteria; GAS5, growth arrest-specific 5; HLA-G,

human leukocyte antigen-G; HSP, heat shock protein; IBD, inflammatory bowel disease; ICAM, intercellular adhesion molecule; IEC, intestinal epithelial cell; IFN-γ, gamma interferon; IL, interleukin; KLF, Krüppel-like factor; lncRNA, long none coed RNA; MAPK, mitogen-activated protein kinase; MDSC, myeloid-derived suppressor cell; miRNA, microRNA; MyD88, myeloid differentiation factor 88; NF-κB, nuclear factor-kappaB; NK, natural killer; PrPC, cellular prion protein; TJ, tight junction; Tregs, regulatory T cell; TG2, transglutaminase type 2; TNF, tumor necrosis factor; UC, ulcerative colitis; VSMCs, vascular smooth muscle cells

References

[1] L. Chen, W. Yang, Y. Guo, W. Chen, P. Zheng, J. Zeng, and W. Tong, Exosomal lncRNA GAS5 regulates the apoptosis of macrophages and vascular endothelial cells in atherosclerosis. Plos One 12 (2017) e0185406.

[2] M. Lucafo, A. Di Silvestre, M. Romano, A. Avian, R. Antonelli, S. Martelossi, S. Naviglio, A. Tommasini, G. Stocco, A. Ventura, G. Decorti, and S. De Iudicibus, Role of the Long Non-Coding RNA Growth Arrest-Specific 5 in Glucocorticoid Response in Children with Inflammatory Bowel Disease. Basic Clin Pharmacol Toxicol 122 (2018) 87-93.

[3] C. Wang, C. Zhang, L. Liu, A. Xi, B. Chen, Y. Li, and D. Jie, Macrophage-Derived mir-155-Containing Exosomes Suppress Fibroblast Proliferation and Promote Fibroblast Inflammation during Cardiac Injury. Molecular Therapy the Journal of the American Society of Gene Therapy 25 (2017) 192.

[4] Y. Xu, B. Zhang, X. Zhu, C. Li, W. Song, and H. Tang, miR155-deficient alleviated intestinal inflammation by downregualting the development and function of CD103+CD11b+DC in the lamina propria of intestine. The Journal of Immunology 198 (2017) 62.8-62.8.

[5] B. Zheng, W.N. Yin, T. Suzuki, X.H. Zhang, Z. Yu, L.L. Song, L.S. Jin, Z. Hong, Z. Hong, and J.S. Li, Exosome-Mediated miR-155 Transfer from Smooth Muscle Cells to Endothelial Cells Induces Endothelial Injury and Promotes Atherosclerosis. Molecular Therapy the Journal of the American Society of Gene Therapy 25 (2017) 1279.

[6] M. Alexander, R. Hu, M.C. Runtsch, D.A. Kagele, T.L. Mosbruger, T. Tolmachova, M.C. Seabra, J.L. Round, D.M. Ward, and R.M. O'Connell, Exosome-delivered microRNAs modulate the inflammatory response to endotoxin. Nature Communications 6 (2015) 7321.

[7] T. Nata, M. Fujiya, N. Ueno, K. Moriichi, H. Konishi, H. Tanabe, T. Ohtake, K. Ikuta, and Y. Kohgo, MicroRNA-146b improves intestinal injury in mouse colitis by activating nuclear factor-κB and improving epithelial barrier function. Journal of Gene Medicine 15 (2013) 249-260.

[8] T. Cooks, I.S. Pateras, L.M. Jenkins, K.M. Patel, A.I. Robles, J. Morris, T. Forshew, E. Appella, V.G. Gorgoulis, and C.C. Harris, Mutant p53 cancers reprogram macrophages to tumor supporting macrophages via exosomal miR-1246. Nature Communications 9 (2018) 771.

[9] G.W. Krissansen, Y. Yang, F.M. Mcqueen, E. Leung, D. Peek, Y.C. Chan, C. Print, N. Dalbeth, M.

Williams, and A.G. Fraser, Overexpression of miR-595 and miR-1246 in the sera of patients with active forms of inflammatory bowel disease. Inflammatory Bowel Diseases 21 (2015) 520.

[10] B. Lönnerdal, X. Du, Y. Liao, and J. Li, Human milk exosomes resist digestion in vitro and are internalized by human intestinal cells. Faseb Journal 29 (1 supplement) (2015) 121.3.

[11] T. Tian, Z. Yu, F. Xiao, S. Ye, W. Hao, W. Wu, W. Tan, C. Yu, J. Hu, and Z. Rong, MicroRNA-16 is putatively involved in the NF-κB pathway regulation in ulcerative colitis through adenosine A2a receptor (A2aAR) mRNA targeting. Scientific Reports 6 (2016) 30824.

[12] T. Wolf, S.R. Baier, and J. Zempleni, The Intestinal Transport of Bovine Milk Exosomes Is Mediated by Endocytosis in Human Colon Carcinoma Caco-2 Cells and Rat Small Intestinal IEC-6 Cells. Journal of Nutrition 145 (2015) 2201-2206.

[13] C.G. Chapman, and J. Pekow, The emerging role of miRNAs in inflammatory bowel disease: a review. Therap Adv Gastroenterol 8 (2015) 4-22.

[14] N. Almási, A. Posa, A. Al-Awar, S. Török, Z. Murlasits, L.I. Nagy, L.G. Puskás, and K. Kupai, Differentially expressed microRNAs and their relation to gasotransmitters in TNBS-induced colitis in rat colon. Academia Journal of Scientific Research 5 (2018) 277-289.

[15] M. Eoin, D. Viola, M. Joanne, B. Kelley, J. Paul, A. Carol, G. Mark, F. Glenn, and E. Holger, P-198 CCR2+ Inflammatory Monocytes Dictate the Course of Colonic Inflammation Via miR-223 Regulation of the NLRP3 Inflammasome. Inflammatory Bowel Diseases 20 Suppl 1 (2014) S105.

[16] J. Li, M. Tan, Q. Xiang, Z. Zhou, and H. Yan, Thrombin-activated platelet-derived exosomes regulate endothelial cell expression of ICAM-1 via microRNA-223 during the thrombosis-inflammation response. Thrombosis Research 154 (2017) 96.

[17] Y. Liao, X. Du, J. Li, and B. Lonnerdal, Human milk exosomes and their microRNAs survive digestion in vitro and are taken up by human intestinal cells. Molecular nutrition & food research 61 (2017).

[18] F. Zaharie, M.S. Muresan, B. Petrushev, C. Berce, G.A. Gafencu, S. Selicean, A. Jurj, R. Cojocneanu-Petric, C.I. Lisencu, and L.A. Pop, Exosome-Carried microRNA-375 Inhibits Cell Progression and Dissemination via Bcl-2 Blocking in Colon Cancer. Journal of Gastrointestinal & Liver Diseases Jgld 24 (2015) 435.

[19] W. Sakamoto, T. Masuno, H. Yokota, and T. Takizawa, Expression profiles and circulation dynamics of rat mesenteric lymph microRNAs. Molecular Medicine Reports 15 (2017) 1989-1996.

[20] Z. Bian, L. Li, J. Cui, H. Zhang, Y. Liu, C.Y. Zhang, and Z. Ke, Role of miR-150-targeting c-Myb in colonic epithelial disruption during dextran sulphate sodium-induced murine experimental colitis and human ulcerative colitis. Journal of Pathology 225 (2011) 544-553.

[21] Y.T. Fang, C.F. Lin, C.Y. Wang, R. Anderson, and Y.S. Lin, Interferon-γ stimulates p11-dependent surface expression of annexin A2 in lung epithelial cells to enhance phagocytosis. Journal of Cellular Physiology 227 (2012) 2775-2787.

[22] S. Tanida, T. Mizoshita, K. Ozeki, T. Katano, H. Kataoka, T. Kamiya, and T. Joh, Advances in refractory ulcerative colitis treatment: A new therapeutic target, Annexin A2. World J Gastroenterol 21 (2015) 8776-8786.

[23] A. Zelante, R. Borgoni, C. Galuppi, V. Cifala, L. Melchiorri, S. Gullini, O. Baricordi, and R. Rizzo, Therapy modifies HLA-G secretion differently in Crohn's disease and ulcerative colitis patients. Inflamm Bowel Dis 17 (2011) E94-5.

[24] V. Rebmann, L. Konig, S. Nardi Fda, B. Wagner, L.F. Manvailer, and P.A. Horn, The Potential of HLA-G-Bearing Extracellular Vesicles as a Future Element in HLA-G Immune Biology. Frontiers in immunology 7 (2016) 173.

[25] M. Burke, W. Choksawangkarn, N. Edwards, S. Ostrandrosenberg, and C. Fenselau, Exosomes from Myeloid-Derived Suppressor Cells Carry Biologically Active Proteins. Journal of proteome research 13 (2014) 836-43.

[26] S.T. Leach, Z. Yang, I. Messina, C. Song, C.L. Geczy, A.M. Cunningham, and A.S. Day, Serum and mucosal S100 proteins, calprotectin (S100A8/S100A9) and S100A12, are elevated at diagnosis in children with inflammatory bowel disease. Scandinavian journal of gastroenterology 42 (2007) 1321-1331.

[27] L. Diaz-Hidalgo, S. Altuntas, F. Rossin, M. D'Eletto, C. Marsella, M.G. Farrace, L. Falasca, M. Antonioli, G.M. Fimia, and M. Piacentini, Transglutaminase type 2-dependent selective recruitment of proteins into exosomes under stressful cellular conditions. Biochimica et biophysica acta 1863 (2016) 2084-92.

[28] L. Elli, M.M. Ciulla, G. Busca, L. Roncoroni, C. Maioli, S. Ferrero, M.T. Bardella, A. Bonura, R. Paliotti, C. Terrani, and P. Braidotti, Beneficial effects of treatment with transglutaminase inhibitor cystamine on the severity of inflammation in a rat model of inflammatory bowel disease. Laboratory investigation; a journal of technical methods and pathology 91 (2011) 452-61.

[29] N. De Silva, M. Samblas, J.A. Martinez, and F.I. Milagro, Effects of exosomes from LPS-activated macrophages on adipocyte gene expression, differentiation, and insulin-dependent glucose uptake. Journal of physiology and biochemistry 74 (2018) 559-568.

[30] J. Caradec, G. Kharmate, E. Hosseini-Beheshti, H. Adomat, M. Gleave, and E. Guns, Reproducibility and efficiency of serum-derived exosome extraction methods. Clinical biochemistry 47 (2014) 1286-92.

[31] H. Ryu, E. Gounaris, M. Kron, C. Waltenbaugh, Z.J. Zhang, and T.A. Barrett, Novel Pathway for TNF-Mediated Albumin Losses in IBD. Gastroenterology 140 (2011) S-502-S-502.

[32] K. Assi, S. Patterson, D.A. Owen, S. Dedhar, M.K. Levings, and B. Salh, Integrin-Linked Kinase Promotes Colitis Through Regulation of CCL2, Fibronectin and Regulatory T Cells. Gastroenterology 140 (2011) S-636-S-636.

[33] A. Purushothaman, S.K. Bandari, J. Liu, J.A. Mobley, E.E. Brown, and R.D. Sanderson, Fibronectin on the Surface of Myeloma Cell-derived Exosomes Mediates Exosome-Cell Interactions. The Journal of biological chemistry 291 (2016) 1652-63.

[34] I. Kurose, S. Miura, M. Suematsu, H. Serizawa, F. Dai, H. Asako, T. Hibi, and M. Tsuchiya, Tissue-type plasminogen activator of colonic mucosa in ulcerative colitis. Digestive Diseases & Sciences 37 (1992) 307-11.

[35] K. Al-Nedawi, J. Szemraj, and C.S. Cierniewski, Mast cell-derived exosomes activate endothelial cells to secrete plasminogen activator inhibitor type 1. Arteriosclerosis, thrombosis, and vascular biology 25 (2005) 1744-9.

[36] A.T. Xu, J.T. Lu, Z.H. Ran, and Q. Zheng, Exosome in intestinal mucosal immunity. Journal of gastroenterology and hepatology 31 (2016) 1694-1699.

[37] Z. Li, M. Zhuang, L. Zhang, X. Zheng, P. Yang, and Z. Li, Acetylation modification regulates GRP78 secretion in colon cancer cells. Scientific Reports 6 (2016) 30406.

[38] I. Das, C.W. Png, I. Oancea, S.Z. Hasnain, R. Lourie, M. Proctor, R.D. Eri, Y. Sheng, D.I. Crane, T.H. Florin, and M.A. McGuckin, Glucocorticoids alleviate intestinal ER stress by enhancing protein

folding and degradation of misfolded proteins. The Journal of experimental medicine 210 (2013) 1201-16.
[39] C. Robertson, S.A. Booth, D.R. Beniac, M.B. Coulthart, T.F. Booth, and A. McNicol, Cellular prion protein is released on exosomes from activated platelets. Blood 107 (2006) 3907-11.
[40] C.S. Petit, F. Barreau, L. Besnier, P. Gandille, B. Riveau, D. Chateau, M. Roy, D. Berrebi, M. Svrcek, P. Cardot, M. Rousset, C. Clair, and S. Thenet, Requirement of cellular prion protein for intestinal barrier function and mislocalization in patients with inflammatory bowel disease. Gastroenterology 143 (2012) 122-32 e15.
[41] S. Da Silva, A.V. Keita, S. Mohlin, S. Pahlman, V. Theodorou, I. Pahlman, J.P. Mattson, and J.D. Soderholm, A Novel Topical PPARgamma Agonist Induces PPARgamma Activity in Ulcerative Colitis Mucosa and Prevents and Reverses Inflammation in Induced Colitis Models. Inflamm Bowel Dis 24 (2018) 792-805.
[42] C. Looze, D. Yui, L. Leung, M. Ingham, M. Kaler, X. Yao, W.W. Wu, R.F. Shen, M.P. Daniels, and S.J. Levine, Proteomic Profiling of Human Plasma Exosomes Identifies PPARγ as an Exosome-associated Protein. Biochem Biophys Res Commun 378 (2009) 433-438.
[43] P. Samborski, and M. Grzymislawski, The Role of HSP70 Heat Shock Proteins in the Pathogenesis and Treatment of Inflammatory Bowel Diseases. Advances in clinical and experimental medicine : official organ Wroclaw Medical University 24 (2015) 525-30.
[44] A. Asea, M. Rehli, E. Kabingu, J.A. Boch, O. Baré, P.E. Auron, M.A. Stevenson, and S.K. Calderwood, Novel Signal Transduction Pathway Utilized by Extracellular HSP70 ROLE OF Toll-LIKE RECEPTOR (TLR) 2 AND TLR4. Journal of Biological Chemistry 277 (2002) 15028-15034.
[45] Z. Deng, J. Mu, M. Tseng, B. Wattenberg, X. Zhuang, N.K. Egilmez, Q. Wang, L. Zhang, J. Norris, and H. Guo, Enterobacteria-secreted particles induce production of exosome-like S1P-containing particles by intestinal epithelium to drive Th17-mediated tumorigenesis. Nature Communications 6 (2015) 6956.
[46] T. Skotland, K. Sandvig, and A. Llorente, Lipids in exosomes: Current knowledge and the way forward. Progress in Lipid Research 66 (2017) 30.
[47] Y. Xiong, Y. Lou, H. Su, Y. Fu, and J. Kong, Cholecalciterol cholesterol emulsion ameliorates experimental colitis via down-regulating the pyroptosis signaling pathway. Experimental & Molecular Pathology 100 (2016) 386-392.
[48] K. Arai, Y. Mizobuchi, Y. Tokuji, K. Aida, S. Yamashita, M. Ohnishi, and M. Kinoshita, Effects of Dietary Plant-Origin Glucosylceramide on Bowel Inflammation in DSS-Treated Mice. Journal of Oleo Science 64 (2015) 737-742.
[49] L.J. Saubermann, P. Beck, Y.P. De Jong, R.S. Pitman, M.S. Ryan, H.S. Kim, M. Exley, S. Snapper, S.P. Balk, S.J. Hagen, O. Kanauchi, K. Motoki, T. Sakai, C. Terhorst, Y. Koezuka, D.K. Podolsky, and R.S. Blumberg, Activation of natural killer T cells by alpha-galactosylceramide in the presence of CD1d provides protection against colitis in mice. Gastroenterology 118 (2000) 119-128.
[50] H.C. Rath, A. Sturm, I. Melchner, J. Schoelmerich, and A.U. Dignass, Proinflammatory effect of lysophosphatic acid on chronic colitis in two animal models of inflammatory bowel disease. Gastroenterology 118 (2000) A1365-A1365.
[51] W. Stremmel, U. Merle, A. Zahn, F. Autschbach, U. Hinz, and R. Ehehalt, Retarded release phosphatidylcholine benefits patients with chronic active ulcerative colitis. Gut 54 (2005) 966-71.
[52] A. Fischbeck, K. Leucht, I. Freywagner, S. Bentz, T. Pesch, S. Kellermeier, M. Krebs, M. Fried, G.

Rogler, and M. Hausmann, Sphingomyelin induces cathepsin D-mediated apoptosis in intestinal epithelial cells and increases inflammation in DSS colitis. Gastroenterology 138 (2011) S-132-S-132.

[53] Phosphatidylinositol signalling defects implicated in IBD. Disease Models & Mechanisms 7 (2014) 1.

[54] M. Vashisht, P. Rani, S.K. Onteru, and D. Singh, Curcumin Encapsulated in Milk Exosomes Resists Human Digestion and Possesses Enhanced Intestinal Permeability in Vitro. Appl Biochem Biotechnol 183 (2017) 993-1007.
